# Microscopic mechanisms of deformation transfer in high dynamic range branched nanoparticle deformation sensors

**DOI:** 10.1038/s41467-018-03396-5

**Published:** 2018-03-20

**Authors:** Shilpa N. Raja, Xingchen Ye, Matthew R. Jones, Liwei Lin, Sanjay Govindjee, Robert O. Ritchie

**Affiliations:** 1 Materials Sciences Division, Lawrence Berkeley National Laboratory, Berkeley, CA 94720 USA; 2 Department of Materials, Science and Engineering, University of California at Berkeley, Berkeley, CA 94720 USA; 3 Department of Chemistry, University of California at Berkeley, Berkeley, CA 94720 USA; 4 Department of Mechanical Engineering, University of California at Berkeley, Berkeley, CA 94720 USA; 5 Department of Civil and Environmental Engineering, University of California at Berkeley, Berkeley, CA 94720 USA; 6 Present Address: Department of Materials Science and Engineering, Massachusetts Institute of Technology, Cambridge, MA 02139 USA; 7 Present Address: Department of Chemistry, Indiana University-Bloomington, Bloomington, IN 47405 USA; 8 Present Address: Department of Chemistry, Rice University, Houston, TX 77251 USA

## Abstract

Nanoscale stress sensing is of crucial importance to biomechanics and other fields. An ideal stress sensor would have a large dynamic range to function in a variety of materials spanning orders of magnitude of local stresses. Here we show that tetrapod quantum dots (tQDs) exhibit excellent sensing versatility with stress-correlated signatures in a multitude of polymers. We further show that tQDs exhibit pressure coefficients, which increase with decreasing polymer stiffness, and vary >3 orders of magnitude. This high dynamic range allows tQDs to sense in matrices spanning >4 orders of magnitude in Young’s modulus, ranging from compliant biological levels (~100 kPa) to stiffer structural polymers (~5 GPa). We use ligand exchange to tune filler-matrix interfaces, revealing that inverse sensor response scaling is maintained upon significant changes to polymer-tQD interface chemistry. We quantify and explore mechanisms of polymer-tQD strain transfer. An analytical model based on Mori-Tanaka theory presents agreement with observed trends.

## Abstract (web summary)

Tetrapod quantum dots are promising nanoscale stress sensors because of their non-invasive sensing technique, but sensor versatility has not yet been assessed. Here the authors show that tetrapod quantum dots exhibit a large dynamic range and excellent sensing versatility in a multitude of polymers.

## Introduction

Nanoscale stresses play a crucial role in a wide variety of fields and processes, such as polymer dynamics and deformation^1^, crack initiation and propagation^2^, and biological processes such as stem cell differentiation. In filler-containing polymers, sensing of nanoscale stresses is of key importance to understanding nanoscale mechanisms of mechanical reinforcement^3,4^ and assessing nanoscale filler-matrix interfacial load transfer^4,5^. Further, it is important for ensuring reproducible, tailored material synthesis^4^. In order to study such nanoscale stresses, an appropriate tool is needed that is sensitive, versatile, and does not alter the properties of the host matrix^4,6^. Further, the ideal nanoscale stress sensor should exhibit stress sensitivity over a large dynamic range, enabling it to detect equally well kilopascal (kPa) stresses in biological systems as well as larger megapascal (MPa) stresses in structural materials.

Current techniques^7,8^ for examining such nanoscale stresses such as Raman spectroscopy^5^, mechanochromic gels^9^, atomic force microscopy (AFM)^10^, electronic skins^11^, metal nanoparticle chains^12^, stress-sensitive small molecules^13^, and others^9^ have limitations, which constrain their utility in practical situations^4,6–8,14^. These include being invasive, having low signal-to-noise ratio, or being limited to specific laboratory settings, material systems, or geometries. Further limitations are that they generally do not exhibit tunable stress sensitivity or survive multiple cycles of stress sensing.

A nanoscale sensor that could report nanoscale stresses in a variety of materials, without the abovementioned limitations, would be highly desirable^4,6,8^. One nanoscale sensor that could serve this purpose is the tetrapod quantum dot (tQD)^15^. tQDs are core-shell cadmium selenide-cadmium sulfide (CdSe-CdS) quantum dots in which the ~4 nm CdSe core has four ~25 nm tetrahedrally branched CdS arms, and exhibits type-I band alignment^15^. The tQD has been shown to be a sensor of local deformation states due to its unique morphology, in which the four tQD arms act as antennae that transmit local deformation to the CdSe core^4,6,8,16,17^. Its branched geometry also makes it an optimal mechanical filler^18^. The tQD response to certain types of stresses consists of a reduction in the bandgap, or a photoluminescence (PL) red-shift, arising from the widening of bonds in the CdSe core^6,17^.

Previous work on tQDs has demonstrated sensing of both extension and contraction^6,17^, sensing of complex behavior such as stress relaxation and hysteresis^4^, sensing of direct contact between adjacent tQDs^6^, and the ability to survive many sensing cycles^6^. However, sensor versatility has not been specifically assessed, as prior studies were limited to only a few polymer host matrices^4,6,8^. Furthermore, studies were only performed with native ligands, i.e., no ligand exchange was performed to change tQD surface chemistry and alter the interfacial strength between the tQD and polymer matrix^4,6,8^. To rectify this situation, we report here on a study using a very wide selection of host materials with varied interfacial conditions.

An assessment of load or strain transfer from the matrix to the filler phase at the nanoscale is also critically important for material systems with embedded sensors^19,20^. However, quantification of strain transfer efficiency in such systems poses a formidable experimental challenge, especially at the nanoscale^5,21^. Probing strain transfer efficiency with tQDs could potentially provide a new unique experimental way to obtain and quantify interfacial dynamics and strain transfer.

Here we build on previous studies by systematically assessing the tQD sensing behavior in a variety of materials with embedded tQDs under tensile stress. The diverse materials studied here comprise eight host matrices and multiple tQD-polymer interfacial chemistries, including tQDs coated with native ligands^15^ and thiol-terminated polymers^22^. We show the ability of the tQD to provide useful response even when embedded in material systems whose Young’s moduli vary by over three orders of magnitude. Intriguingly, more compliant, or lower stiffness, polymers result in a monotonically higher stress response sensitivity, or pressure coefficient (defined as change in tQD PL bandgap in meV per GPa of applied stress^23^). In the most compliant polymers, the tQD pressure coefficients are orders of magnitude higher than in bulk CdSe^23^. The tQD is a highly versatile sensor with a large dynamic range, enabling it to detect low kPa stresses for biomechanical applications as well as orders of magnitude higher stresses (MPa level) in stiffer structural materials. We propose that this high dynamic range originates from varying degrees of strain transfer at the critical polymer-tQD interface due to the tQD’s branched geometry. Further, in a unique use of visible light experiments to elucidate polymer-filler strain transfer dynamics^5,19,24,25^, we determine the strain transfer efficiency across the tQD-polymer interface from our in situ stress measurements. A unique corollary of this feature is that it allows us to assess the validity of classical self-consistent micromechanical theories on strain concentration tensors^26–28^.

## Results

### Material system preparation

In this work, we studied 17 material systems, which included fibers and films, as well as a variety of different tQD concentrations and dispersions in multiple tQD-ligand-polymer systems. The systems in this work vary widely in terms of interfacial chemistry between polymer and tQD, polymer composition and hydrophobicity, and mechanical properties, with Young’s moduli varying more than four orders of magnitude across all host matrices in this work.

The tQDs were synthesized using established methods^15^. As-synthesized tQDs were incorporated into polymer fibers or films with either their native ODPA ligands or after ligand exchange to coatings of thiol-terminated polymers^22^, such as poly-l-lactide (PLLA), to create a stronger polymer-tQD interfacial bond. The polymers used in this work included poly(ethylene oxide) (PEO), polycaprolactone (PCL), poly(styrene-ethylene-butylene-styrene) (SEBS), PLLA, polydimethylsiloxane (PDMS), and polybutadiene (PBD)^29–31^. Electrospinning was used for PLLA, PEO, SEBS, and PCL; a viscous polymer chloroform solution was mixed with a chloroform solution of nanoparticles to create viscous solutions of 4–12% by weight polymer, and 0.05–20% by weight/0.01–5% by volume tQDs; droplets of the highly viscous solution were subject to high electric fields (15 kV/cm) to form aligned arrays of fibers using the dual-rod geometry of Li et al.^4,32^. Single fibers were collected from aligned arrays for optical and mechanical tests. Electrospun fibers were examined with an optical microscope to measure their diameters and morphologies, and were seen to generally have a smooth, uniform appearance (Supplementary Fig. 1). Films were prepared by mixing solutions of polymer, nanoparticles, and chloroform into glass vials and then drying them in air or under streams of nitrogen^6^. PBD fibers were hand-drawn from viscous PBD-chloroform solutions.

**Fig. 1:**
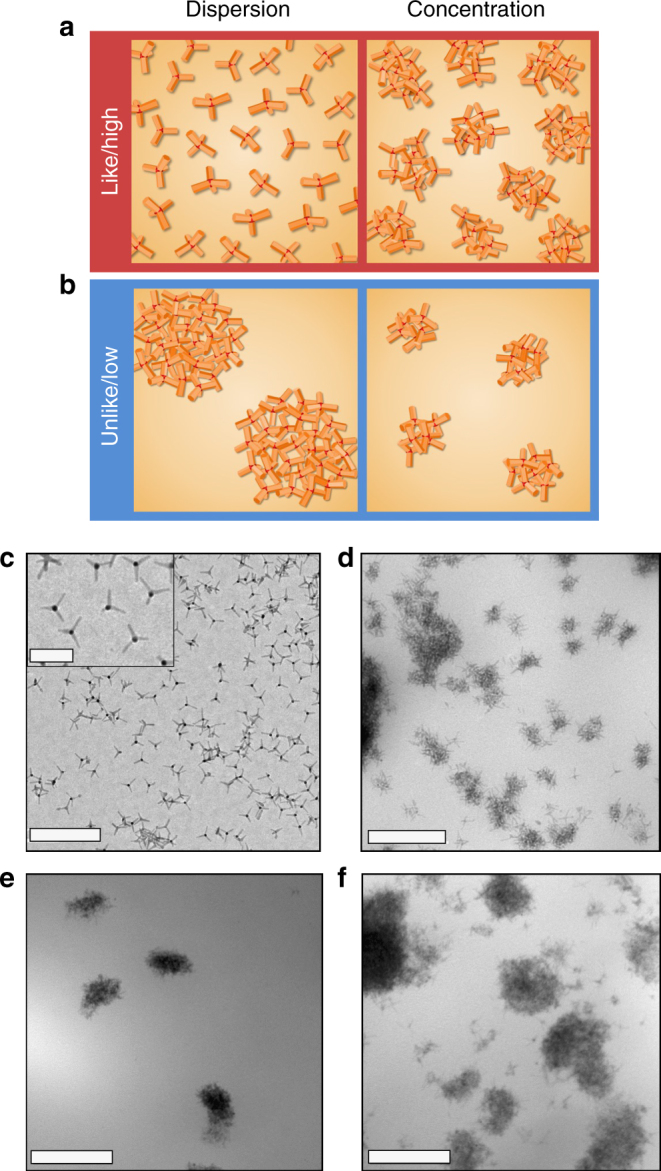
tQD cluster dispersion and concentration in polymers and tQD-polymer interfaces studied in this work (see Fig. 2 for evenly dispersed tQDs in polymers). **a**, **b** Schematics of high (evenly dispersed) and low (clustered) dispersions are shown, as well as high and low concentrations in polymers wherein tQDs form clusters, and varying tQD-polymer interface chemistries. **c** TEM images of tQDs before polymer incorporation. **d** TEM image of tQD clusters in PLLA (10% by weight). **e** TEM image of tQD clusters in SEBS (5% by weight). **f** TEM image of tQD clusters in SEBS (20% by weight). All scale bars shown represent 200 nm, except for the inset to **c**, which is 50 nm

**Fig. 2:**
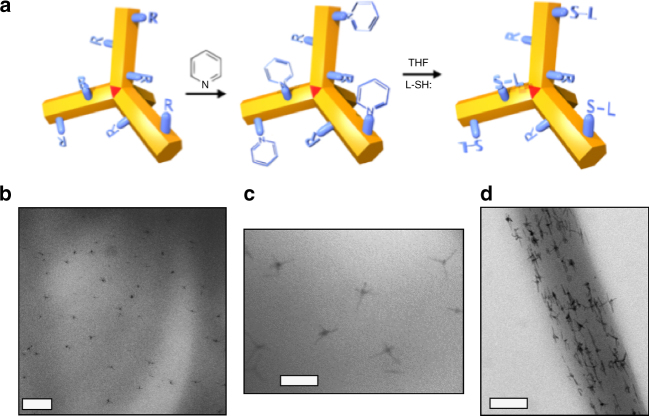
Singly dispersing tQDs into polymers. **a** Schematic of the two-step process to coat tQDs with thiol-terminated polymers. **b**, **c** TEM images of PLLA-coated tQDs in PLLA polymer. **c** Closer view. **d** TEM image of PLLA-coated tQDs in PEO fiber. Scale bars are **b** 200 nm; **c** 40 nm; and **d** 80 nm

Figure 1 shows transmission electron microscopy (TEM) images of some of the material systems with embedded tQDs studied in this work. It illustrates the clustering seen in the native ligand-tQD systems due to the chemical incompatibility between the host matrix and the hydrophobic ODPA tQD native ligands^18,20,30,33^. On increasing concentration in these systems, the tQD cluster size grew slightly while the cluster spacing reduced (Fig. 1). Images of evenly or singly dispersed tQD-polymer systems can be found in Fig. 2, and additional TEM images of more of the polymers can be found in Supplementary Fig. 1.

Note that tQDs do not exhibit a preferred orientation in the polymers as a function of drawing. While drawing at high strains can result in orientation of nanorods^31^, it has no effect on the orientation of tQD fillers. This is due to the tetrahedral symmetry of the tQD^15^, e.g., the tQDs in Fig. 2b–d of the main text were drawn to over a thousand percent via hand-drawing or electrospinning, but no preferred orientation was seen.

Nanoparticle surface ligand density, chemistry, and molecular weight are known to affect polymer matrix-nanoparticle interactions. Such factors can affect nanoparticle dispersion in a polymer matrix, as well as other properties^22,34,35^. In particular, ligands with similar composition to the polymer host matrix, as well as longer lengths and intermediate grafting density, have been shown to result in better dispersions. Motivated by these findings and the fact that tQDs with native short alkyl chain ligands aggregate in all polymer matrices studied (Fig. 1, Supplementary Fig. 1), we coated them with polymer ligands at intermediate grafting density. We did this via ligand exchange of tQDs to long-chain (~2.5 kDa) polymeric coatings. Our goal was to improve tQD dispersion to look at its effect on sensing behavior. The exchange was done using a two-step process, from the tQD’s native octadecylphosphonic acid^15^ to pyridine and then to a polymer ligand layer^22^, in order to study the effect of interfacial polymer-tQD chemical bond strength on the embedded tQD pressure coefficient^20^. By introducing thiol-terminated PLLA (SH-PLLA) and SH-PEO onto tQD surfaces, we achieved a good dispersion, free of aggregation, into these polymers (Fig. 2). tQDs coated with SH-PLLA dispersed well into both PEO and PLLA, with individual tQDs seen in the polymer matrix in TEM images (Fig. 2). This significantly improved dispersion is indicative of a stronger like-like interface between the tetrapod and polymer^22^. Nuclear magnetic resonance (NMR) showed that ~60% of the ligands on the tQD surface were thiol-terminated polymeric ligands, with a surface ligand density^40^ of ~2/nm^2^, within the range of previous findings for CdSe quantum dots^41^ (Supplementary Fig. 2). Despite this notably improved interfacial interaction, dynamic scanning calorimetry measurements did not reveal any change in glass transition temperature, *T*_g_^4^, likely because the tQD concentration was too small to impact *T*_g_ (0.2% or less by volume).

### tQD sensing versatility and high dynamic range

For a nanosensor probe to be of use for a broad spectrum of applications, versatility of sensing response in a wide variety of host matrices is essential. Additionally, a large dynamic range of pressure coefficient is highly desirable^23^. For example, in biological settings, stresses between cells or between cell and substrate are often on the order of kPa^42^, while in structural applications, stresses can be on the order of MPa^43^. These applications require very different pressure coefficients—if the same pressure coefficient was used in biological applications as for structural parts, the optical shifts corresponding to the stresses applied to tQDs would be two orders of magnitude too low to resolve on any commercially available charge-coupled devise (CCD) detector. Thus, the tQD sensor response in both relatively stiff and compliant host materials needs to be assessed.

**Fig. 3:**
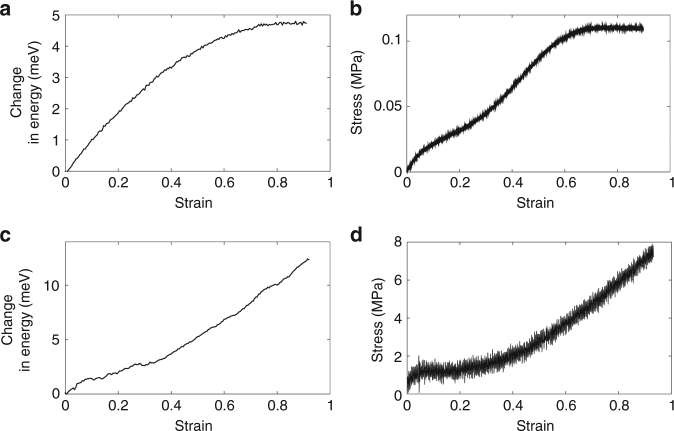
Examples of opto-mechanical and mechanical tests on tQD-PBD and tQD-PCL polymer fibers. Opto-mechanical and mechanical data were acquired separately. **a** Optically sensed fluorescence tensile curve of tQD-PBD fibers. The *x*-axis is tensile strain, while the *y*-axis is the magnitude of the PL emission maximum red-shift. **b** Corresponding tensile stress–strain curve of tQD-PBD fibers measured using a typical uniaxial mechanical tensile tester. **c** Optically sensed fluorescence tensile curve of tQD-PCL fibers. The *y*-axis is the magnitude of the PL emission maximum red-shift. **d** Corresponding tensile stress–strain curve of tQD-PCL fibers measured using a typical uniaxial mechanical tensile tester. Engineering stress and engineering strain are plotted

**Fig. 4:**
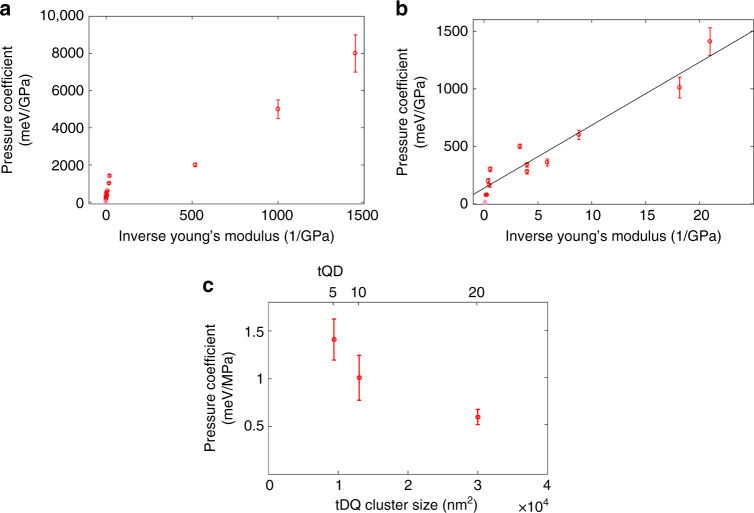
High dynamic range of tQD pressure coefficients. **a** Monotonic scaling of the tQD pressure coefficient over three orders of magnitude with the polymer inverse Young’s modulus. **b** Plot of the initial region shown in **a** (inverse Young’s modulus <200/GPa). **c** Plot of pressure coefficient as a function of tQD cluster size for three concentrations of tQDs in SEBS. Error bars represent standard error of the mean (SEM) and each mean is the average of 10–15 measurements

Accordingly, after sample preparation, the tQD sensor response was assessed via optical spectroscopy while applying tensile strain to the polymers (see Methods). This was done using a mechanical stretcher with a hole for laser passage^4,6^. Raw spectra as a function of stretching were collected at quasi-static strain rates, and then fit to single Gaussians to determine the PL emission maximum (i.e., the peak of the PL emission spectrum) as a function of stretching (Supplementary Fig. 3). Traditional mechanical testing was conducted using a spring-based load cell (Mark-10) for films or with an Agilent T-150 nanomechanical tensile testing machine (see Methods). All material systems studied in this work exhibited clear tQD spectral red-shifts upon tensile extension (Fig. 3, Supplementary Fig. 3), demonstrating clear versatility of the tQD sensor response in a wide variety of material systems. Typical optical and mechanical measurements for tQD-PCL and tQD-PBD fibers are shown in Fig. 4. For fibers, mechanical and opto-mechanical tests were performed separately, while for films they were performed simultaneously (see Methods).

We examined trends in the tQD pressure coefficient as a function of several variables. These included polymer modulus, tQD aggregation, tQD concentration, and tQD-polymer interfacial interactions.

**Fig. 5:**
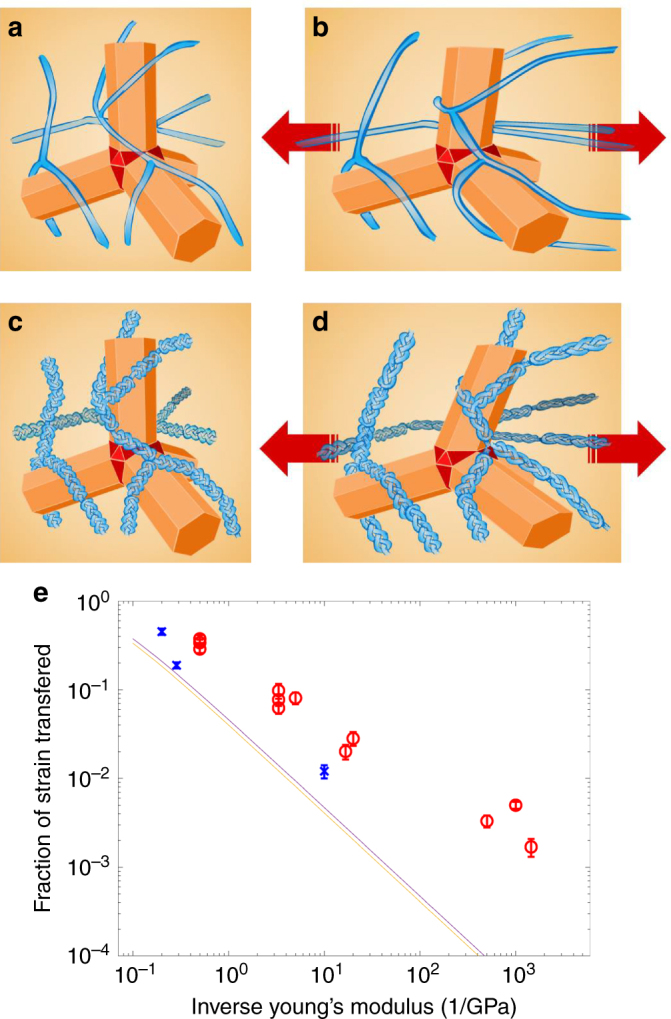
Polymer-tQD strain transfer efficiency. **a** Schematic of tQD-polymer interfacial strain transfer. **a**, **b** Lower strain transfer efficiency from relatively low Young’s modulus polymers to the tQDs, while **c** and **d** depict higher strain transfer efficiency from relatively high Young’s modulus (higher stiffness depicted as analogous to a braid) polymers to tQDs. **e** Red circles and blue Xs indicate experimental values. Blue Xs represent systems with varied ligand-tQD surface chemistry. Polymer-tQD strain transfer efficiency as a function of inverse Young’s modulus of the polymer material. The plot shows lines that indicate our theoretical Mori-Tanaka model predictions; the yellow line represents the theoretical model with a polymer Poisson’s ratio of 0.3, while the purple line represents the theoretical model with a polymer Poisson’s ratio of 0.5. Error bars represent SEM and each mean is the average of 10–15 measurements

We uniquely found an enhancement of up to more than three orders of magnitude in the tQD pressure coefficient with decreasing polymer Young’s modulus (Fig. 5). All materials exhibited tQD PL energy gap red-shifts of 2–15 meV upon uniaxial stretching, even those with orders of magnitude lower stresses. In the most compliant polymers, this corresponds to an amplification of the tQD pressure coefficient over bulk CdSe by 100–300 times^23,44^. This means that tQDs are able to report stresses effectively both in stiff structural and compliant polymer materials, implying that tQDs are ideal nanoscale probes with high dynamic range.

In Fig. 5, the standard error in the calculation of the pressure coefficient from experimentally measured red-shift and stress is of similar percentage for each system. We also included pressure coefficients from previous studies utilizing preformed polyester fibers in which tQDs were incorporated via diffusion, and tQDs in frozen toluene in diamond anvil cells^8,17^.

### Possible origins of large tQD pressure coefficient range

Potential factors responsible for the observed tQD pressure coefficient amplification and wide dynamic range include the following: (i) changes in tQD dispersion in the host matrix; (ii) changes in the tQD-polymer interface chemistry; (iii) amplification of stress around the tQD in the polymer due to the tQD arms’ nanoscale size and thus relative sharpness^45^; and (iv) varying degrees of strain transfer from the polymer to the tQD. As described below, we conclude that a potential explanation for this phenomenon is a molecular-scale polymer chain-tQD interaction leading to strain transfer efficiency that monotonically varies with the polymer Young’s modulus, as has been observed previously^46^.

Regarding changes in tQD dispersion, we have determined that this is an unlikely cause for the observed phenomena by using TEM image analysis to compute average aggregate sizes and aggregate packing fractions for polymers that contained tQD clusters (Fig. 4c, Supplementary Fig. 4). We found no trend in the pressure coefficient with varying tQD aggregate size, tQD aggregate packing density, or tQD concentration in a given polymer matrix except for one system, tQD-SEBS. In this case the pressure coefficient varied by a factor of only two, as compared to more than three orders of magnitude through varying the host matrix Young’s modulus (Fig. 4). In addition, statistical analyses showed much higher correlations for Young’s modulus and pressure coefficient than any of the other abovementioned variables (see Methods and Supplementary Fig. 4). Thus, changes to tQD dispersion are not likely to be major causal factors for the observed pressure coefficient trends (see Methods for adjusted *R*-squared values).

The second hypothesis was that interface strength and pressure coefficient would monotonically scale because of more strain transfer to tQDs at stronger interfaces. Interestingly, however, this also fails to explain the observed results, because the tQD pressure coefficient is not seen to scale with increasing tQD-polymer interfacial strength. While the vast majority of systems studied in this work showed mostly little to no change between the Young’s modulus of the pure polymer and the tQD-polymer (Supplementary Fig. 5), evenly dispersing tQDs by coating them with PLLA (Fig. 3) increased the polymer elastic modulus by 25–75% (Supplementary Fig. 6). However, the pressure coefficient simultaneously decreased by 50–75%. This is consistent with our finding that stiffer polymers have decreased tQD pressure coefficients (Fig. 4).

Stress concentration at the tQD arm tips and edges due to their sharpness^47,48^ was eliminated as a mechanism for the observed results of pressure coefficient amplification in more compliant polymers. This is because such amplification is localized to the tip of the arm and is far from the tQD core^47,48^.

We suggest a likely explanation is that the tQD fillers in different polymer matrices are experiencing different degrees of strain transfer from the polymer matrix^46^. This has a literature precedent because stress or strain transfer efficiencies from polymers to embedded fibers have been found to vary monotonically with the ratio of the Young’s modulus of the stiff fiber and more compliant polymer^46^. This leads to pressure coefficient amplification in lower stiffness materials because of the monotonically varying Young’s modulus difference between the stiff tQD and increasingly more compliant host polymer, and lower stress in lower stiffness polymers^46,49^. Stress amplification is known to occur in biological materials when a very more compliant material is bound to a very stiff material and the more compliant material is strained, causing the stiffer material to bear full or partial equal strain and thus higher stress^50^. Such a situation is often encountered in bone implants, when strain transfer from the surrounding compliant bone to the much stiffer implant problematically leads to stress amplification in the implant phase (stress shielding)^49^.

A very simple, qualitative, elastic model can be used to evaluate tQD-filler strain transfer efficiencies. An elastic model is appropriate because owing to its branched shape and partial strain transfer from the polymer, it is likely that the tQD deformation during mechano-optical stress sensing is fully elastic^4,6,8,17^. Previous studies have shown that due to their unique core-arm bending modes, tQDs remain elastic to more than 30% bending strain as imposed by an AFM tip^16^. Furthermore, upon the removal of stress to the polymers, the tQDs always return to the same baseline fluorescence peak position, and have excellent cyclability of the sensing upon repeated deformation^4,6^, a strong indicator that tQD deformation remains elastic throughout the polymer tensile test^17^.

As a very basic model, one can consider the load on the tQD-polymer system to be carried by the polymer and tQDs acting in parallel. In the elastic regime and in the limit of very strong binding, the strains will be equal in both the polymer matrix and the tQD-filler phase of the material, *ε*_t_ =* ε*_p_. Further assuming elastic response of the components gives the tQD stress as $\sigma _{\mathrm{t}} = \frac{{\sigma _{\mathrm{p}}E_{\mathrm{t}}}}{{E_{\mathrm{p}}}}$, where *E*_t_ and *E*_p_ are the Young’s moduli of the tQD and polymer, respectively, and *σ*_p_ is the polymer stress. The amplification effect is thus seen, in this very simple model, to be proportional to the ratio of the Young’s moduli^50^.

However, the geometry of the tQDs in the polymer matrix makes this very simple parallel model rather unrealistic; additionally, the binding between the two is unlikely to be as perfect as the model assumes. Molecular dynamics studies have shown that polymer chains tend to wrap conformally around spherical and elongated nanoparticles^51^. In the case of tQDs and polymers, the interface likely consists of polymer chains wrapped around individual and/or multiple tQD arms, likely forming multiple loops^51^ (Fig. 5a–d). Such molecular-level wrapping between the polymer and tQDs is an important effect that contributes to tQD-polymer strain transfer efficiency, and thus to the observed results, but this is impossible to capture in numerical models using present-day computers. Further, this wrapping is again unlikely to create a strong enough interface to achieve equal strain in the tQD and polymer phases of the material. Accordingly, our simple parallel element model will likely overpredict the experimentally observed tQD stresses and pressure coefficients.

However, due to the branched morphology and multiple bending modes of the tetrapod^18^, it is perhaps conceivable that this wrapping could lead to bending of tQD arms and partial strain transfer from the polymer to the tQD core at the arm-core junctions^16^. Its branching could give rise to a knotting of multiple chains around the tQD (Fig. 5a–d), so that entangled chains as a collective could transfer deformation and hence strain to the tQD.

We note that while we did observe stress relaxation in most systems (Supplementary Fig. 3)^4,6^, such stress relaxation does not lead to any changes in pressure coefficient. This is because the mechanical stress and optical shift decay similarly during stress relaxation^4,6^.

### Polymer matrix-tQD-filler strain transfer efficiency

To investigate the partial strain transfer in our tQD-polymer materials, we introduced a phenomenological parameter *x* for the degree of strain transfer, *ε*_t_ = *xε*_p_. Assuming elasticity as before, allows us to express the degree of strain transfer as:

1 $$x = \frac{{\sigma _{\mathrm{t}}E_{\mathrm{p}}}}{{\sigma _{\mathrm{p}}E_{\mathrm{t}}}}.$$

The stress in the tQD, *σ*_t_, can be determined by dividing the optical stress shifts for each system by the tQD pressure coefficient, as was assessed in prior work using diamond anvil cells^17^. For the tQD Young’s modulus, *E*_t_, we used the value for bulk CdS^52^. The polymer stress, *σ*_p_, and Young’s modulus, *E*_p_, were determined using uniaxial tensile testing. Figure 5e shows a plot of the fraction of strain transfer from the polymer matrix to the tQD across the tQD-polymer interface as a function of host matrices’ Young’s moduli. Data are shown for all systems that we studied experimentally. The plot indicates that tQDs experience increasing strain transfer as the ratio of tQD Young’s modulus to host matrix modulus is reduced; i.e., the more compliant materials have lower strain transfer efficiency.

As in previous studies^4,6,8,17^, we did not see any degradation in the mechanical properties upon incorporation of tQDs at any of the concentrations used in this work, which ranged from 0.05 to 20% by weight, or 0.01 to 5% by volume. This is true in cases where tQDs did not affect the mechanical properties^4^, as well as in cases of like-like interfaces where tQDs enhanced the Young’s modulus (Supplementary Fig. 6). This indicates that tQDs are not acting as damage initiators.

We note that the above assumption of elasticity is reasonable even for the more rubbery polymers considered in this work, PDMS and high-molecular-weight SEBS and PBD. Various types of PDMS, for instance, have been reported to be highly elastic rather than viscoelastic^53^. Similarly, PBD has been shown to exhibit high elasticity^54^. Also, our previous work showed that SEBS is highly elastic.^29^. In spite of their high elasticity, the systems do have a relatively small component of viscoelasticity, which leads to relaxation observed in the first 100 s or so in the stress relaxation modulus for PDMS^55^. This is consistent with our observation of stress relaxation for most of the systems (Supplementary Fig. 3)^4,6^. However, all of our pressure coefficient measurements were conducted after 200 s, meaning that the systems had already relaxed and that stress relaxation is not affecting our pressure coefficient measurement.

We modeled the stress response and dynamic range of the tQD material systems utilizing a model that we developed from the Mori-Tanaka theory for the mechanics of heterogeneous material systems, a field approximation method based on the Eshelby model (details are described in the Mori-Tanaka analysis section in Methods below)^26–28^. This second model was developed in order to validate the new simple uniaxial model (Eq. 1). We present our Mori-Tanaka theoretical model for two host matrix Poisson’s ratios that fall within the boundaries of the material systems in this work. Despite the fact that the Mori-Tanaka theory makes a number of simplifying assumptions, such as linear elasticity of the system, and uniform dispersion of the tQDs in all polymers, it is seen to qualitatively match the experimental results. In addition to validating existing micromechanical theories^26–28^, this finding corroborates our simple uniaxial model (Eq. 1), showing that despite the complexity of our tQD-polymer systems, their behavior can be qualitatively captured using simple models. This result further indicates that the tQD response remains elastic even at large deformations in the lowest stiffness materials studied here.

We note that despite the fact that Mori-Tanaka theory was originally developed for non-interacting fillers, multiple studies have successfully applied it for non-dilute cases, even as high as 10–50% by volume^36–38,56^. In this work, as evidenced by the TEM images in Figs. 1 and 2 and Supplementary Fig. 1, tQDs are interacting, with one exception being the evenly dispersed system in Fig. 2b. However, in all cases, we are below the 10–50% limit since the maximum volume fraction that we consider is 5%. Regardless, the fact that the theory was originally developed for non-interacting systems may contribute to the fact that only qualitative agreement is seen.

Figure 5a–d schematically illustrate our finding that tQDs wrapped with lower Young’s modulus materials shift less than tQDs wrapped with stiffer polymers. Strain transfer efficiencies range from 0.2% for the most compliant to 45% for the stiffest polymers that we tested. The findings of this work are consistent with previous reports indicating that filler-polymer load transfer efficiency decreases as the Young’s modulus of the host matrix is decreased^46^. In spite of this lower strain transfer in lower Young’s modulus polymers, we find them to have a higher tQD pressure coefficient. This is because even though there is less strain transferred to tQDs in relatively compliant matrix materials, the stress in the polymer is far lower, which results in an overall higher pressure coefficient. (see Supplementary Figs 7 and 8 for plots of stress transfer efficiencies with respect to changes in host material Young’s modulus, according to our Mori-Tanaka model).

## Discussion

In this work, we have employed experimental opto-mechanical and mechanical characterization to demonstrate that branched tQDs exhibit excellent stress sensing capabilities to track mechanical stress–strain behavior in a wide spectrum of polymeric systems. Specifically, we have studied 17 tQD-polymer systems covering over eight polymer host matrices, multiple tQD-polymer interfacial chemistries, several tQD concentrations and dispersions, and more than four orders of magnitude variation in host matrix Young’s modulus. We find that changing the Young’s modulus of the host matrix varies the tQD stress response, or pressure coefficient, by over three orders of magnitude. We present a method to functionalize tQDs with different polymeric ligands, achieving excellent dispersion of tQDs in multiple polymer matrices. Clear, cyclable stress sensing is observed in all polymers, with the mechanical properties of the polymers not degraded at all by the tQD additions. We further determine the strain transfer efficiency from the polymer to the tQD, finding that the efficiency increases for stiffer host matrix materials. Our results also represent a validation of the Mori-Tanaka theory for such systems. These findings indicate the high versatility of tQD stress sensors to a wide range of structural and biomedical applications as well as to fundamental polymer dynamics studies.

## Methods

### Materials

All chemicals were used as received. Chloroform, pyridine, and tetrahydrofuran were purchased from Sigma-Aldrich. PLLA (100 kDa molecular weight) was purchased from ShenZhen ESUN Industrial Co Ltd. SH-PLLA (2.5 kDa molecular weight) was purchased from Polymer Source. SEBS (117 kDa molecular weight, MD-1537) was purchased from Kraton Corporation. Polyethylene oxide (300 kDa molecular weight), PDMS (purchased in monomer form), PCL (80 kDa molecular weight), and PBD (*cis*, 200–300 kDa molecular weight) were purchased from Sigma-Aldrich.

### tQD synthesis

Chemical precursors used were purchased from Sigma-Aldrich. CdSe-CdS core-shell tQDs were synthesized in the absence of moisture and oxygen, using a two-step seeded synthesis method. To start, zinc-blend CdSe seeds were prepared by mixing cadmium myristate with selenium dissolved in octadecene. This mixture was heated to 170 °C, causing the nucleation of CdSe seeds, followed by injection of oleic acid and oleylamine ligands and growth of the CdSe seeds at 240 °C. Next, seeds were cleaned by repeated centrifugation in polar solvents (isopropanol and acetone). Next, wurtzite CdS arms were grown on the cleaned CdSe seeds by syringe-injecting them via airfree transfer into a heated mixture of *n*-propylphosphonic acid, trioctylphosphine, trioctylphosphine oxide, and *n*-cotadecylphosphonic acid. Growth of arms then occurred at 320 °C. The tQDs were then transferred to a glovebox and cleaned via repeated centrifugation in polar solvents. tQD samples in this work had arm lengths ranging from 22 to 29 nm, arm diameters ranging from 4 to 6 nm, and core sizes of 3.5 to 4.5 nm, analyzed from TEM images using ImageJ. Within these tQD size ranges, no statistically significant difference in the optical sensing response was seen in any given nanocomposite system.

### Nuclear magnetic resonance

A Bruker Avance500 II NMR spectrometer system (Bruker, Billerica, MA) was used to conduct NMR spectroscopy. The *δ* scale is utilized to present shifts, and the unit of hertz is used for coupling constants. Deuterated chloroform was used as solvent, while the standard was tetramethylsilane.

### Electrospinning and hand-drawing precursor solutions

Fibers of SEBS, PLLA, PCL, PBD, and PEO were dissolved in chloroform to create solutions of 12%, 12%, 10%, 12%, and 5% by weight, respectively. tQDs were then added in a chloroform solution at a variety of concentrations ranging from 0.05 to 20% by weight, or 0.01 to 5% by volume. SEBS, PLLA, PCL, and PEO were electrospun using the procedure described below, while PBD was hand-drawn with a syringe needle dipped into the viscous polymer solution directly onto mechanical tabs for mechanical tests or directly onto the piezo-drive for optical tests.

### Fiber synthesis

Polymer-tQD fibers were prepared using either electrospinning or hand-drawing. For electrospinning, a droplet of polymer-tQD solution was placed on the end of a syringe needle (Nordson, 38.1 mm/0.51 mm gauge length/inner diameter, part number 7018225) before application of an electric field between the needle and collector, which resulted in fiber formation. As-formed fiber diameters ranged from 1 to 10 μm, and single fibers were formed by employing a dual-rod collector geometry. Eight-millimeter-diameter stainless steel rods were placed 95 mm apart during electrospinning. Fibers were used as-formed. A bias of 15 kV between the needle and collector was used, with a 150 mm separation between needle and collector, resulting in an electric field of 1 kV/cm. For hand-drawing of fibers, highly viscous solutions of polymer in chloroform were used; these were of similar viscosity to the electrospinning solutions. Fibers were manually pulled from the highly viscous solution using a pipette tip before direct deposition onto a tensile testing tab.

### Tensile mechanical testing

Samples were prepared for mechanical tests by directly gluing electrospun fibers to small 5 mm × 10 mm cardboard tabs with diamond central cut-outs for stability. Specialized electrospun fiber transfer tools made of twisted pipe cleaners and carbon tape were used to transfer as-spun fibers to tensile testing tabs. Epoxy glue was used to secure fibers. Tensile mechanical testing using an Agilent T-150 nanomechanical tensile tester was performed at quasi-static strain rates using standard pivot grips, or in a custom-built tensile tester with a hole for laser passage using a Mark-10 0.5 N load cell for SEBS films. PDMS-tQD composites were tested using a custom load frame (Psylotech) in a confocal microscope (WiTEC). Young’s moduli were assessed in the initial linear elastic region of uniaxial tensile stress–strain curves. Each averaged data point for the value of the Young’s modulus for the different polymers and composites represents tests from 5–15 trials. All mechanical tests in this work were conducted at room temperature. All stresses and strains presented are engineering stresses and strains.

### Inverted fluorescence spectroscopy system

The nanocrystal fluorescence was excited with a 488-nm Ar+ laser (Lexel Laser, Inc., 95) with 1 W power and 250 μm spot size at the sample. Bright-field and fluorescence images were taken with a digital microscope camera (Paxcam 2+). The fluorescence spectra were monitored using a custom-built inverted fluorescence microscope with a spectrometer (Acton Research Corporation, SpectraPro-3001) and CCD detector (Princeton Instruments, Model 7509-0001). Exposure times of 1 s were used to collect spectra with a 0.6 s lag time between frames. Spectra were collected and binned over the area of the laser spot and fit to single Gaussians; for local mapping, rows of the camera in groups of threes or fours were binned, rather than binning over the entire laser excitation area on the CCD camera. Change in emission was defined as the difference between the peak position at time *t* and the peak position at zero strain. Stress relaxation rates were determined by fitting the emission shift versus time to a single exponential decay. For mechanical tests, stress was substituted for emission shift.

### Monitoring nanocomposite fluorescence during deformation

To monitor fluorescence while stretching fibers in tension, a piezo-stretcher mounted via screws on a metal platform was used; the platform had a hole to allow the laser to reach the sample. The piezo-drive was controlled with Lab-View. The gauge length for optical tests was 1.8 mm for PLLA and 330 or 500 μm for SEBS, PCL, PEO, and PBD. Fibers were mounted directly onto pieces of tape put onto the arms of the piezo-drive, over which epoxy glue was applied and tests were started 15–30 min later to allow the glue sufficient time to dry. All opto-mechanical tests in this work were conducted at room temperature.

### Characterization of fiber morphology and size

The diameters of tQD-polymer fibers were imaged and photographed using a 63X objective lens on a standard optical microscope (QCapture camera and QImaging software), which was calibrated using a transmission electron microscope grid (11.85 pixels/μm). Fiber diameters ranged from 1 to 10 μm and were analyzed from digital camera images using ImageJ. Film thicknesses ranged from ~150 μm to 1 mm and were assessed using digital calipers with a resolution of 1 μm (Mitutoyo).

### Determination of pressure coefficient

The pressure coefficient was determined by taking the average stress-induced fluorescence red-shift in units of milli-electron volts (meV) across 5–15 optical tests of the nanocomposites at a given engineering strain, and dividing this quantity by the average uniaxial stress in the nanocomposite at the same engineering strain in gigapascals (GPa). In the literature, this is a conventional unit for the pressure coefficient^23^. The point of maximum optical red-shift achievable in our home-built fluorescence tensile stretcher, 2–10 meV depending on the system, was used to determine pressure coefficient. Then to obtain stresses for pressure coefficient determination, the engineering stress at the same engineering strain from mechanical tests was used. This strain ranged from 0.7 to 2. No difference was seen within error in the pressure coefficient for a particular system when evaluated at higher or lower engineering strains. For all systems, the pressure coefficient was evaluated after a minimum of 200 s had passed in the mechanical or opto-mechanical tensile tests. For films, optical and mechanical tests were performed simultaneously, while for fibers, due to their low strength, a highly specialized load cell was used which required doing the two sets of tests on separate instruments. Each pressure coefficient value represents averages from 5 to 15 different trials. The percent covariance (standard deviation divided by the mean) is fairly similar for all composite Young’s moduli and pressure coefficients, which is why the error bar increases with increasing pressure coefficient and composite modulus in Fig. 2 in the main text.

Note that while we observed stress relaxation in nearly all nanocomposite systems^4,6^, such stress relaxation did not affect the pressure coefficient measurement due to similar decay rates for mechanical stress and optical shift during stress relaxation.

### Preparation of nanocomposite films

To prepare SEBS-tQD nanocomposite films, 25 mg of SEBS was dissolved in 2 mL of a tQD-chloroform solution at appropriate concentration to create films of 20% by weight tQDs (5% by volume). These precursor solutions were put into glass vials and dried using a vigorous stream of nitrogen, resulting in film drying occurring within 1–2 min. PDMS films were prepared using a kit with two-part composition (prepolymer base and curing agent) and a 10 to 1 ratio of prepolymer base to curing agent. After mixing, the PDMS was cured at room temperature.

### TEM and sample preparation

For TEM, single fibers or a random fiber network were either deposited directly onto copper TEM grids, or fibers or films were embedded in epoxy and then microtomed at cryogenic temperatures using an ultramicrotome (Boeckler, RMC MT-X). Sections were imaged using a 200 kV Tecnai G2 transmission electron microscope.

### tQD-ligand exchange

tQD-ligand exchange was performed using a two-step procedure. First, tQDs were dissolved in pyridine and then centrifuged using hexane. This process was repeated thrice to replace the native octadecylphosphonic acid coating with pyridine to the greatest extent possible^22^. Next, SH-PLLA dissolved in tetrahydrofuran was mixed with pyridine-coated tQDs, until tQDs solubilized in the tetrahydrofuran, which was used as an indication that the exchange had completed. NMR was then employed to determine the exchange rate of 60% exchange to SH-PLLA.

### Statistical analysis

In order to more quantitatively determine that the Young’s modulus was the main variable correlative with the tQD pressure coefficient, we performed linear fitting to the pressure coefficient as a function of several variables. For both linear regimes shown in Fig. 5, the Young’s modulus is the main correlative variable with the tQD pressure coefficient, with adjusted *R*-squared values of 0.99 and 0.93 for the first and second regimes, respectively. Other dispersion-related nanocomposite variables showed little to no correlation, such as tQD concentration (−0.03 adjusted *R*-squared), tQD aggregate cross-sectional area, as determined by TEM image analysis (0.3788 adjusted *R*-squared), and tQD aggregate packing fraction, also assessed by TEM image analysis (0.3274 adjusted *R*-squared). Respective plots are shown in Supplementary Fig. 4.

### Mori-Tanaka analysis

When the tQDs are well dispersed in a host matrix at a low to modest volume fraction, the average state of stress and strain in the tQD can be understood using the theory of Mori and Tanaka^28^. While this theory is developed for the linear elastic regime^26,27^, it can still be used to qualitatively understand the trends observed in our experiment. From an experimental point of view, we measure several basic quantities: the stress and strain in the composite polymer-tQD system (which we equate to the host polymer stress and strain due to the low loading fractions) and the PL shift in the tQDs. Knowing the pressure coefficient of the tQD, this latter quantity allows us to infer the stress in the tQD. Note that this inference assumes that the pressure coefficient is known for the exact state of stress in the tQD in the host matrix.

The essential result from the theory of Mori and Tanaka^28^ that we use is that the volume average strains in the tQD (t) and the matrix (m) are related by:

2 $$< \varepsilon > _{\mathrm{t}} = {\cal A}: < \varepsilon > _{\mathrm{m}},$$

where the strain concentration tensor for the tQD is given by:

3 $${\cal A} = \left[ {{\Bbb I} + {\Bbb P}:\left( {{\Bbb C}_{\mathrm{t}} - {\Bbb C}_{\mathrm{m}}} \right)} \right]^{ - 1}.$$

In Eq. (3), ${\Bbb I}$ is the symmetric fourth-order identity tensor, ${\Bbb P} = {\Bbb S}:{\Bbb C}_{\mathrm{m}}^{ - 1}$, ${\Bbb C}_{\mathrm{m}}$ is the fourth-order modulus tensor for the host matrix, ${\Bbb C}_{\mathrm{t}}$ is the fourth-order modulus tensor for the tQD, and ${\Bbb S}$ is the interior Eshelby tensor (for which we utilize the solution for a spherical inclusion in an infinite matrix).

The CdSe core has a zinc-blend structure (space group $F\bar 43m$) with anisotropic elastic constants^39^. Given the limitations of our actual knowledge of the precise state of the system, we employ isotropic elastic constants for the CdSe by projecting the full fourth-order elasticity tensor onto the space of isotropic elasticity tensors, viz., $\left\| {{\Bbb C} - {\Bbb C}^{{\mathrm{iso}}}} \right\| \to \min$. This results in a Young’s modulus of *E*_CdSe_ = 44.6 GPa and a Poisson’s ratio of *ν*_CdSe_ = 0.334. The CdS has a wurtzite structure (space group *P*6_3_*mc*) with anisotropic elastic constants^39^. The isotropic projection of these properties results in a Young’s modulus of *E*_CdS_ = 48.3 GPa and a Poisson’s ratio or *ν*_CdS_ = 0.349. Given these projections, it is reasonable to take the Eshelby tensor to be ${\Bbb S} = \frac{2}{3}{\Bbb I}^{{\mathrm{vol}}} + \frac{7}{{15}}{\Bbb I}^{{\mathrm{dev}}}$, where ${\Bbb I}^{{\mathrm{vol}}} = \frac{1}{3}{\mathbf{1}} \otimes {\mathbf{1}}$ is the volumetric fourth-order identity tensor, and ${\Bbb I}^{{\mathrm{dev}}} = {\Bbb I} - {\Bbb I}^{{\mathrm{vol}}}$ is the fourth-order deviatoric identity tensor.

Evaluation of Eq. (3) results in:

4 $${\cal A} = \frac{1}{{\frac{1}{3} + \frac{{2K_{\mathrm{t}}}}{{3K_{\mathrm{m}}}}}}{\Bbb I}^{{\mathrm{vol}}} + \frac{1}{{\frac{8}{{15}} + \frac{{7\mu _{\mathrm{t}}}}{{15\mu _{\mathrm{m}}}}}}{\Bbb I}^{{\mathrm{dev}}},$$

where $K = \frac{E}{{3\left( {1 - 2v} \right)}}$ is the isotropic bulk modulus and $\mu = \frac{E}{{2\left( {1 + v} \right)}}$ is the isotropic shear modulus. If we concern ourselves with the axial strain transfer to the tQD and assume that the matrix deforms in a near incompressible fashion, then the relevant expression for the strain transfer coefficient is:

5 $$< \varepsilon _{11} > _{\mathrm{t}} = x < \varepsilon _{11} > _{\mathrm{m}},$$

6 $$x = {\cal A}_{1111} - \frac{1}{2}{\cal A}_{1122} - \frac{1}{2}{\cal A}_{1133} = \frac{1}{{\frac{8}{{15}} + \frac{{7\mu _{\mathrm{t}}}}{{15\mu _{\mathrm{m}}}}}}.$$

Equation (6) is plotted in Supplementary Fig. 9 over the experimental range of host material compliances (inverse Young’s moduli) at two representative values for the Poisson’s ratio of the host material. The trend qualitatively matches the experimental data and is quantitatively close too, despite being a linear elastic theory with a number of simplifying assumptions. (Note a mean value of *μ*_t_ = 17.5 GPa was used for the plot.)

The low values of the strain transfer coefficient for soft host materials still allow for nontrivial values for the stress transfer coefficient. Within the Mori-Tanaka theory, the stress transfer relation is given by:

7 $$< \sigma > _{\mathrm{t}} = {\cal B}: < \sigma > _{\mathrm{m}},$$

where the stress concentration tensor for the tQD is given by:

8 $${\cal B} = \left[ {{\Bbb I} + {\Bbb Q}:\left( {{\Bbb C}_{\mathrm{t}}^{ - 1} - {\Bbb C}_{\mathrm{m}}^{ - 1}} \right)} \right]^{ - 1} = \frac{{K_{\mathrm{t}}}}{{K_{\mathrm{m}}}}\frac{1}{{\frac{1}{3} + \frac{{2K_{\mathrm{t}}}}{{3K_{\mathrm{m}}}}}}{\Bbb I}^{{\mathrm{vol}}} + \frac{{\mu _{\mathrm{t}}}}{{\mu _{\mathrm{m}}}}\frac{1}{{\frac{8}{{15}} + \frac{{7\mu _{\mathrm{t}}}}{{15\mu _{\mathrm{m}}}}}}{\Bbb I}^{{\mathrm{dev}}}.$$

In Eq. (8), ${\Bbb Q} = {\Bbb T}:{\Bbb C}_{\mathrm{m}}$, and the conjugate Eshelby tensor ${\Bbb T} = {\Bbb I} - {\Bbb C}_{\mathrm{t}}:{\Bbb S}:{\Bbb C}_{\mathrm{t}}^{ - 1}$. Assuming a uniaxial state of stress in the host material, the stress transfer coefficient for the stress component in the tQD in the direction of the load is given by:

9 $$< \sigma _{11} > _{\mathrm{t}} = y < \sigma _{11} > _{\mathrm{m}},$$

10 $$y = {\cal B}_{1111} = \frac{1}{3}\frac{{K_{\mathrm{t}}}}{{K_{\mathrm{m}}}}\frac{1}{{\frac{1}{3} + \frac{{2K_{\mathrm{t}}}}{{3K_{\mathrm{m}}}}}} + \frac{2}{3}\frac{{\mu _{\mathrm{t}}}}{{\mu _{\mathrm{m}}}}\frac{1}{{\frac{8}{{15}} + \frac{{7\mu _{\mathrm{t}}}}{{15\mu _{\mathrm{m}}}}}}.$$

The stress transfer coefficient for the pressure in the tQD is given by:

11 $$< p > _{\mathrm{t}} = y_v < \sigma _{11} > _{\mathrm{m}},$$

12 $$y_v = \frac{1}{3}\left( {{\cal B}_{1111} + {\cal B}_{2211} + {\cal B}_{3311}} \right) = \frac{1}{3}\frac{{K_{\mathrm{t}}}}{{K_{\mathrm{m}}}}\frac{1}{{\frac{1}{3} + \frac{{2K_{\mathrm{t}}}}{{3K_{\mathrm{m}}}}}}.$$

Equation (10) is plotted in Supplementary Fig. 7 over the experimental range of host material compliances (inverse Young’s moduli) at two representative values for the Poisson’s ratio of the host material. Equation (12) is plotted in Supplementary Fig. 8 over the experimental range of host compliances (inverse Young’s moduli) at two representative values for the Poisson’s ratio of the host material.

### Data availability

The authors confirm that all relevant data are available upon reasonable request.

## Electronic supplementary material

Supplementary Information(PDF 897 kb)
