# Supplementary material for: Microscopic mechanisms of deformation transfer in high dynamic range branched nanoparticle deformation sensors
Source: Nat Commun. 2018 Mar 20;9:1155. doi: 10.1038/s41467-018-03396-5 (PMC5861061; doi:10.1038/s41467-018-03396-5)
Supplement: Supplementary file 1 — Supplementary Information(PDF 897 kb) [file 41467_2018_3396_MOESM1_ESM.pdf]

Microscopic Mechanisms of Deformation Transfer in High Dynamic Range Branched Nanoparticle Deformation Sensors

Raja *et al*.

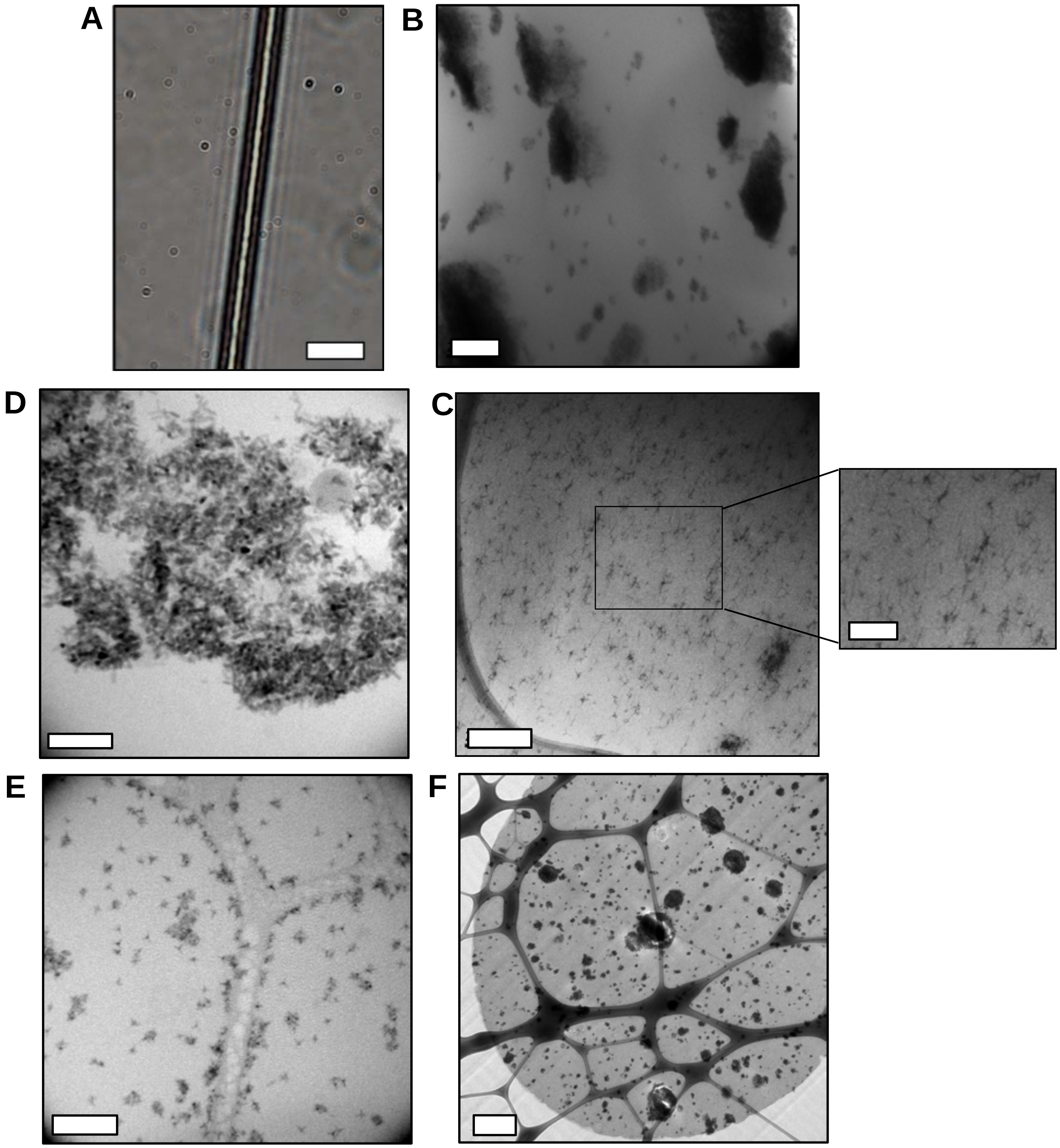

Supplementary Figure 1. Optical Image of Electrospun Fiber and Transmission Electron Microscopy (TEM) Images of Nanocomposites. A. Optical image of electrospun fiber. Scale bar 8 μm. B. TEM image of PCL-tQD nanocomposite, 10% by weight tQD. Scale bar 200 nm. C. TEM image of SEBS-tQD nanocomposite film, 20% by weight tQD Scale bar 100 nm. D. TEM image of PLLA-tQD in PLLA, 0.75% by wt. tQD. Scale bar 200 nm. Inset scale bar 100 nm. E. TEM image of PBD-tQD nanocomposite, 1% by wt. (large curved features are the holey carbon TEM grid). Scale bar 200 nm. F. Large-area view of cryo-microtomed cross-section of 20% by wt. tQD-SEBS nanocomposite fiber. Scale bar 200 nm.

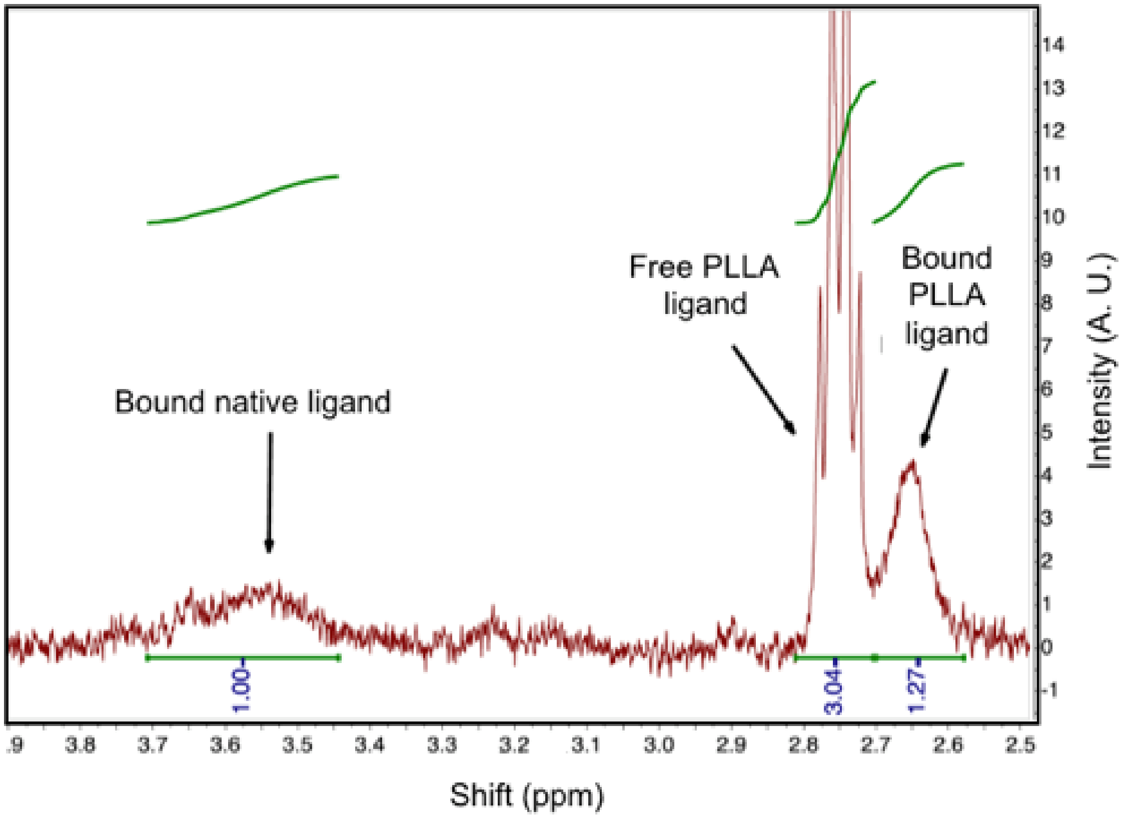

Supplementary Figure 2. NMR spectrum of poly-l-lactide (PLLA)-coated tQDs. Green curved lines represent peaks taken for integration. Green straight lines represent the x-range chosen for integration of the respective peaks. Blue numbers represent the values of the peak integrals.

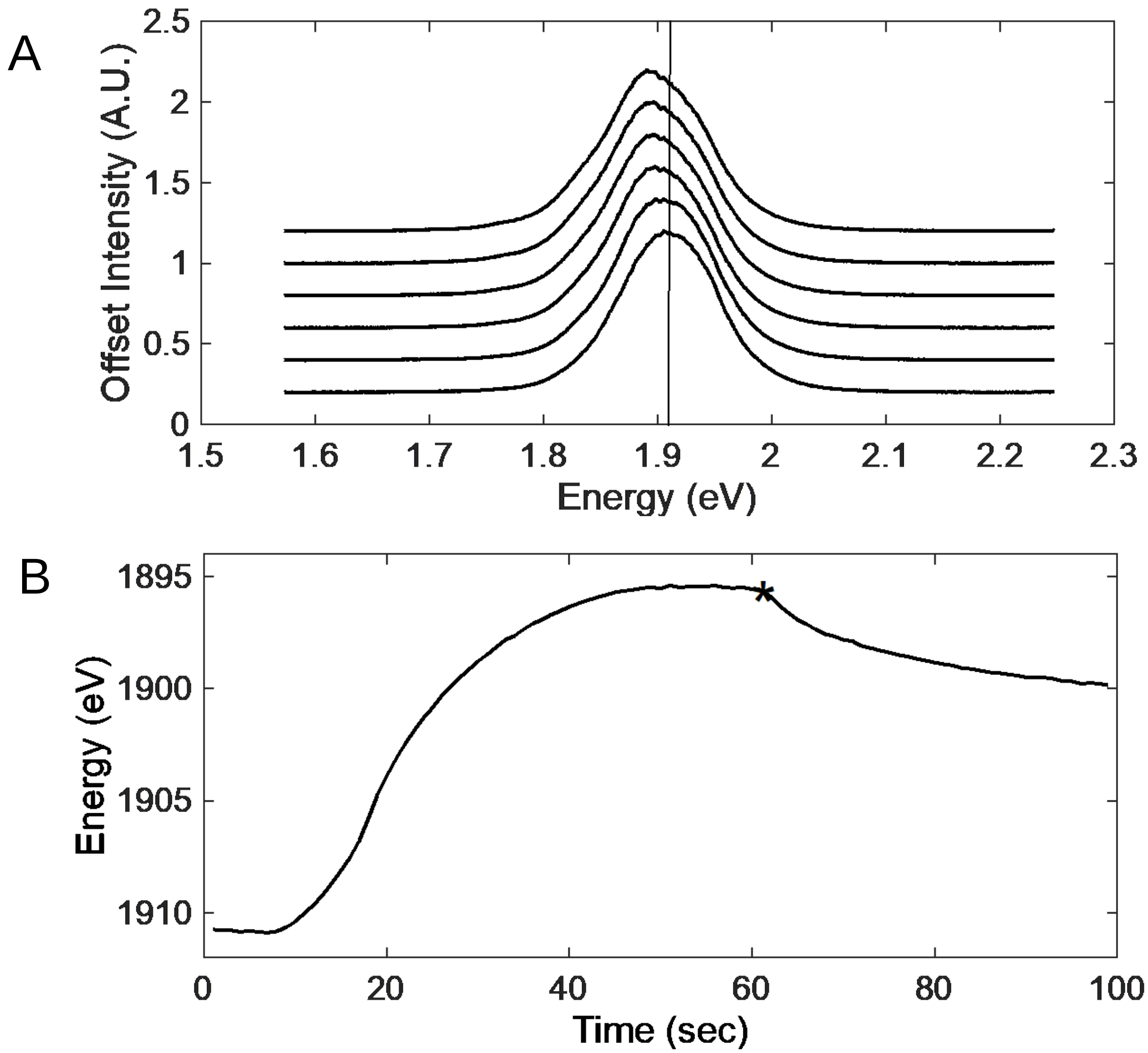

Supplementary Figure 3. Raw spectral peaks and tQD PL emission maximum redshift in evenly dispersed PLLA-tQD-PLLA nanocomposites. A. Sampling of PLLA-tQD-PLLA nanocomposite raw spectral peaks as a function of degree of tensile extension. A clear redshift of the spectral emission maximum is visible. The black line is a guide for the eye. B. Shift in tQD spectral emission maximum during tensile extension, obtained by fitting raw spectral peaks to single Gaussians and plotting the maxima with time. Star indicates holding for stress relaxation.

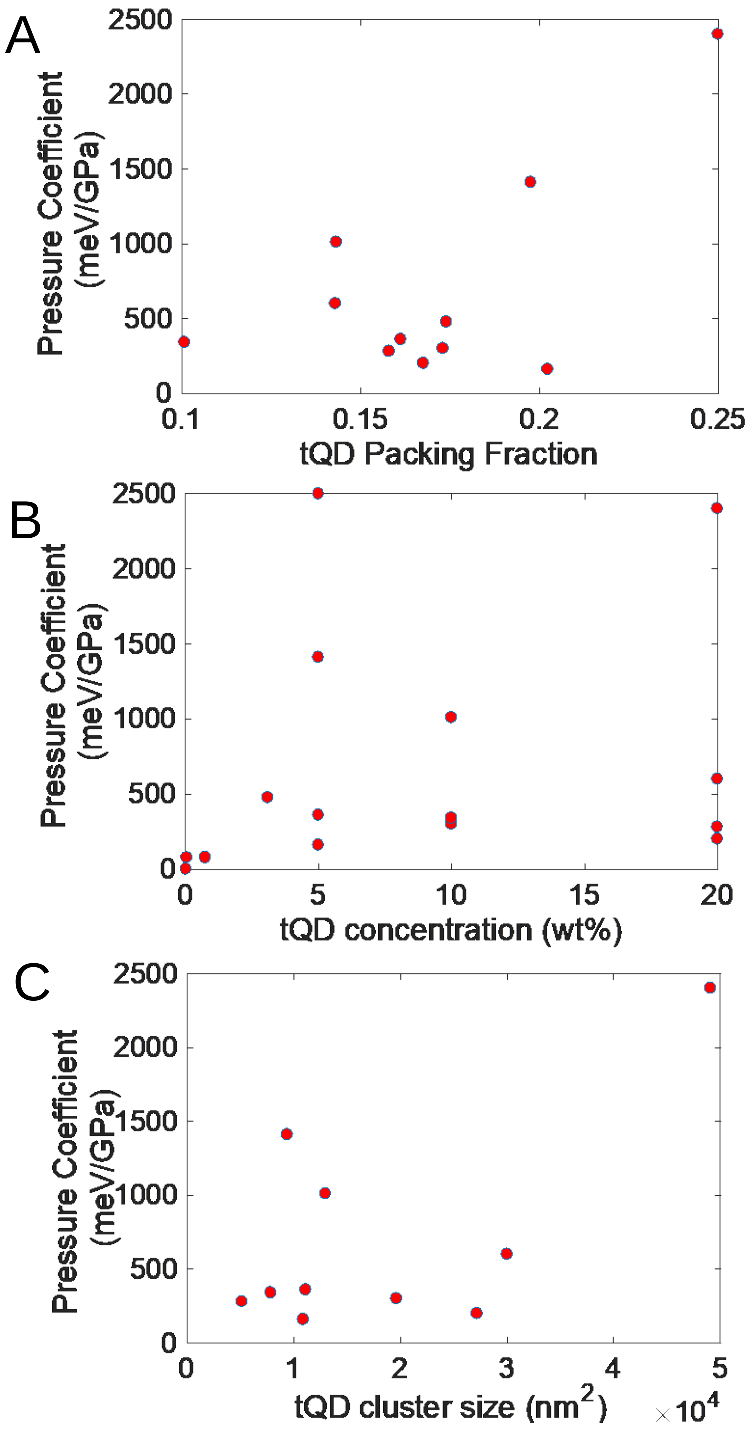

Supplementary Figure 4. Plots of tQD Nanocomposite Pressure Coefficient as a Function of Dispersion-Related, Poorly Correlated Variables. A. tQD nanocomposite pressure coefficient as a function of tQD nanocomposite packing fraction. B. tQD nanocomposite pressure coefficient as a function of tQD concentration. C. tQD nanocomposite pressure coefficient as a function of tQD cluster cross-sectional area.

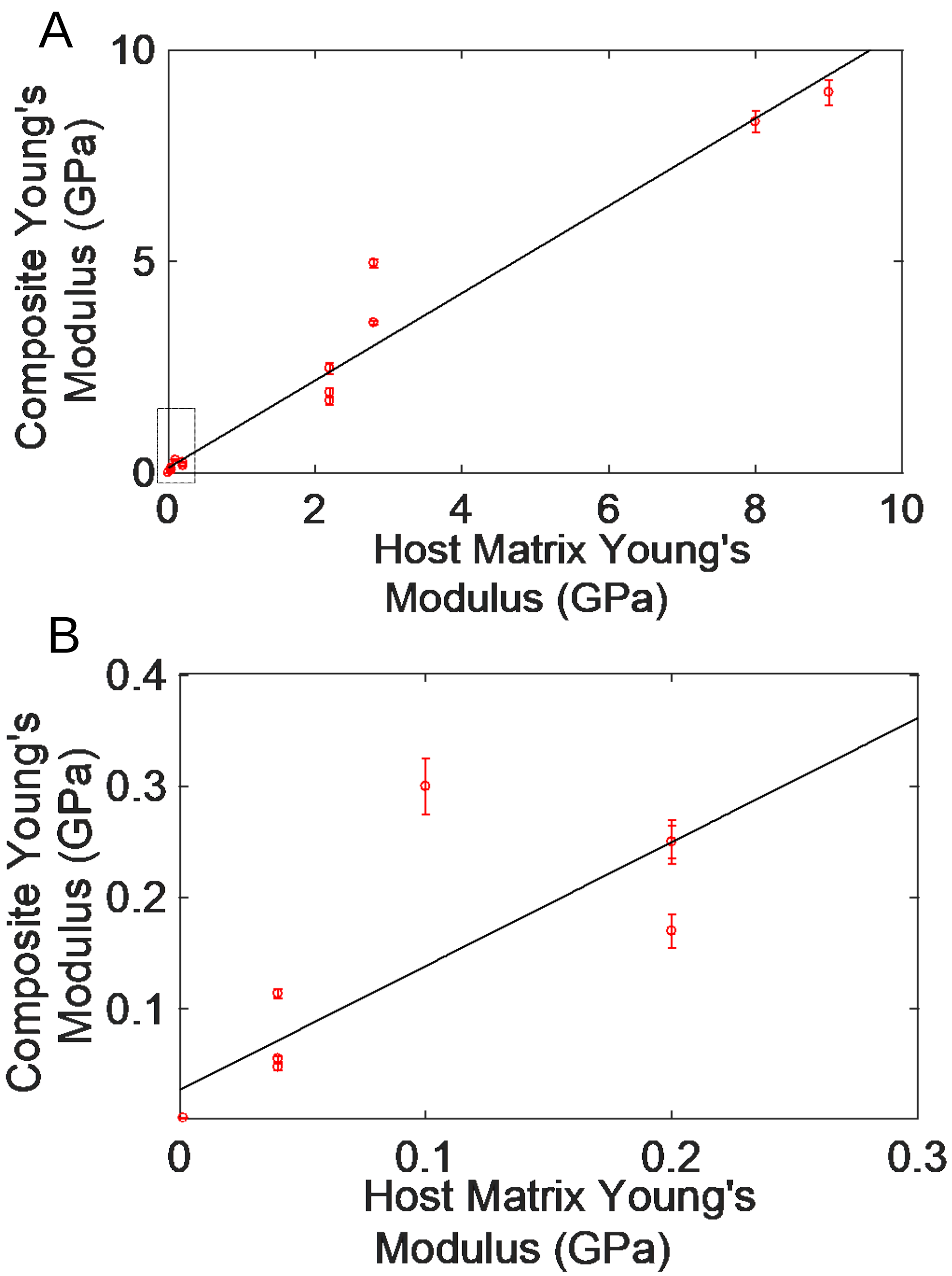

Supplementary Figure 5. Comparison of Young's Modulus of Host Matrix with Young's Modulus of Nanocomposite. A. Host matrix *vs*. nanocomposite Young's modulus. B. Inset to A as indicated by boxed region in A. Error bars represent S.E.M. Each statistic represents measurements on a minimum of 8 samples.

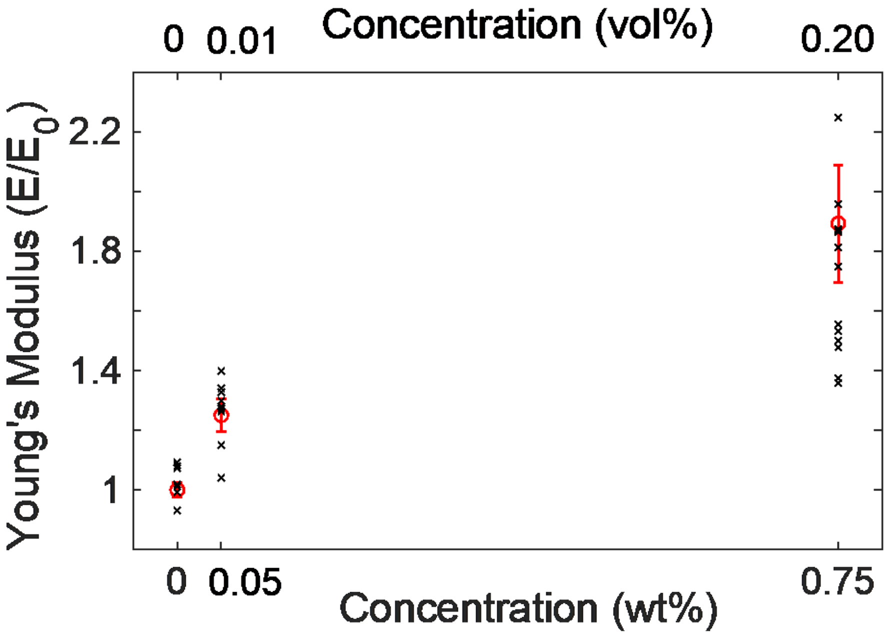

Supplementary Figure 6. Young's Modulus of Evenly Dispersed tQD-PLLA Polymer System. Variation in Young's modulus with tQD concentration in the evenly dispersed PLLA-tQD-PLLA material system. Error bars represent S.D. and each mean is calculated from measurements on 8-12 samples. Black 'x's represent values from individual sample test.

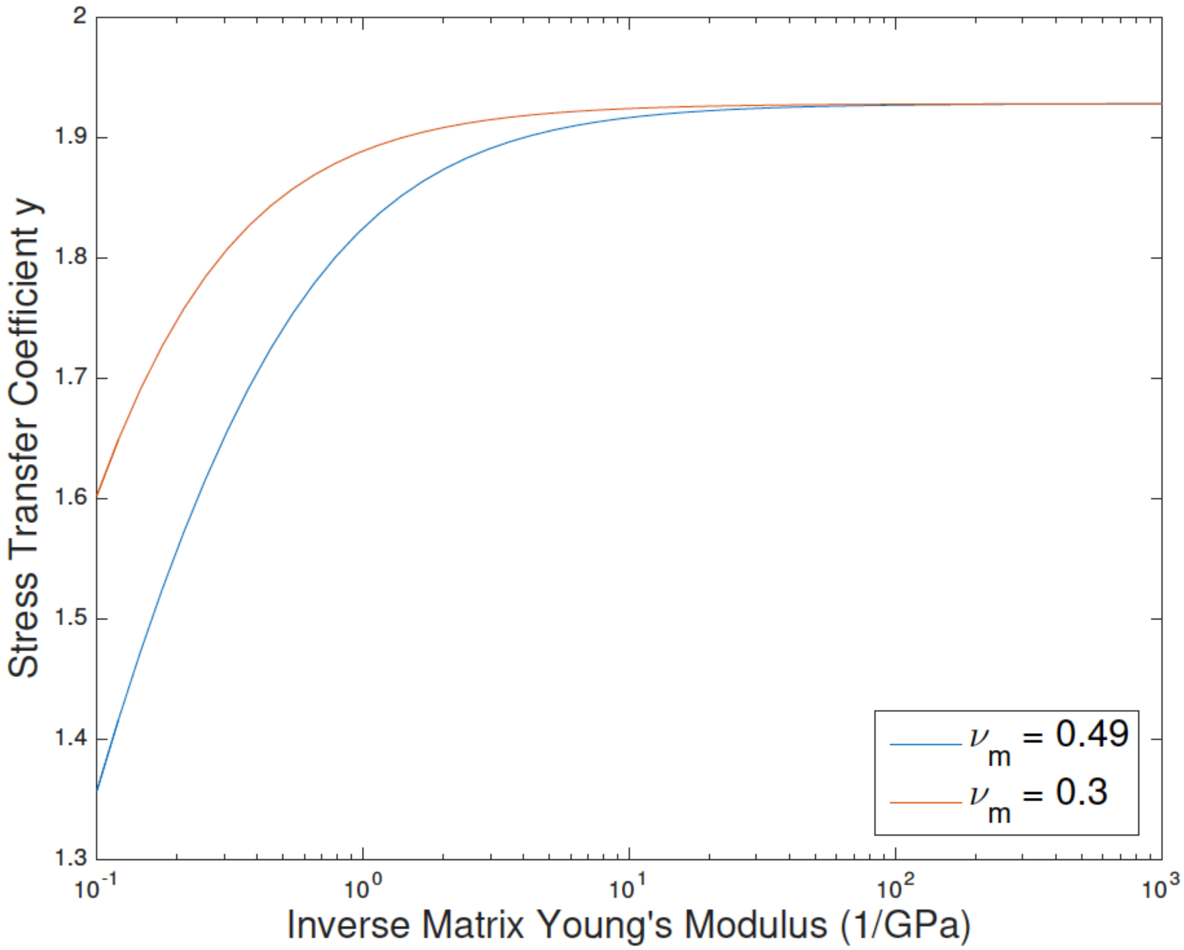

Supplementary Figure 7. Stress Transfer Coefficient *y* for Two Representative Host Material Poisson Ratios.

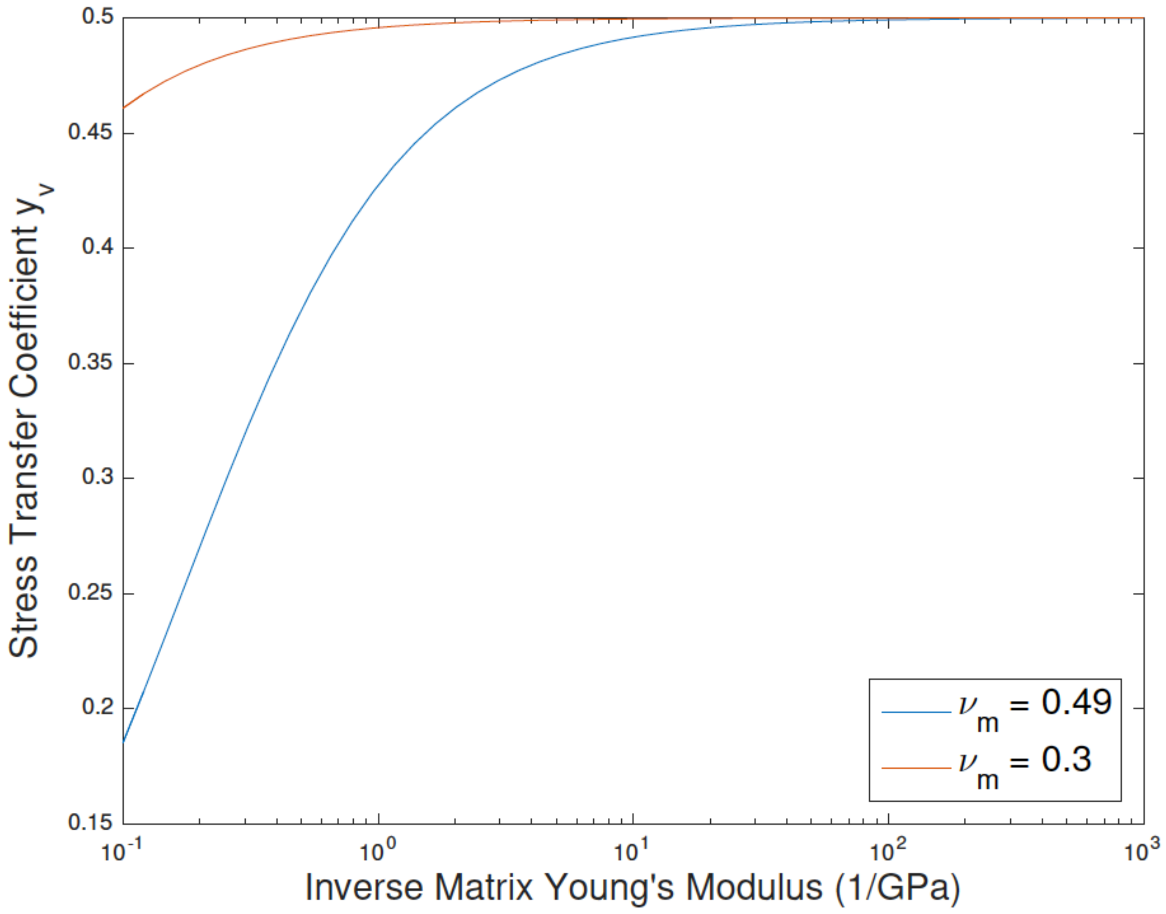

Supplementary Figure 8. Stress (Pressure) Transfer Coefficient y_v for Two Representative Host Material Poisson Ratios.

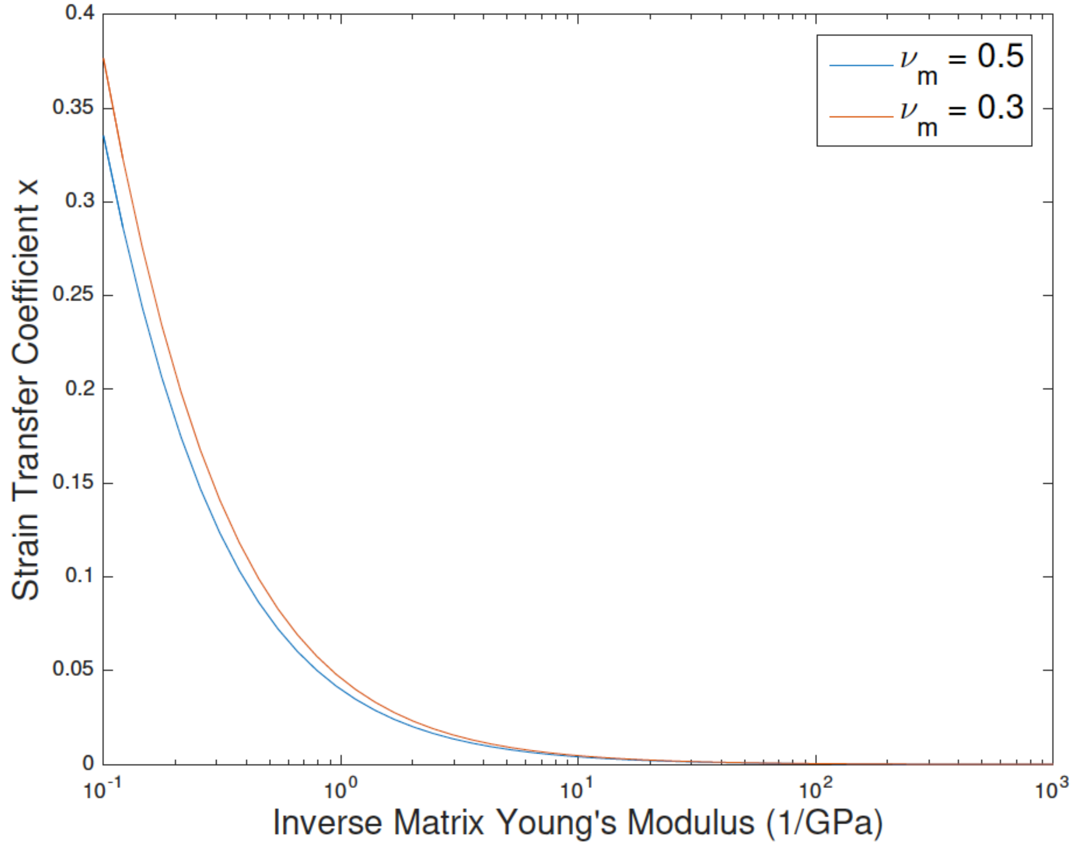

Supplementary Figure 9. Strain Transfer Coefficient *x* for Two Representative Host Material Poisson Ratios.
